# The human right to safely give birth: data from 193 countries show that gender equality does affect maternal mortality

**DOI:** 10.1186/s12884-022-05225-6

**Published:** 2022-11-24

**Authors:** Tanmay Bagade, Catherine Chojenta, Melissa Harris, Christopher Oldmeadow, Deborah Loxton

**Affiliations:** 1 Centre for Women’s Health Research, School of Medicine and Public Health, College of Health, Medicine and Wellbeing, The University of Newcastle (UON), University Drive, Callaghan, NSW 2308 Australia; 2 Hunter Medical Research Institute, Clinical Research Design, IT and Statistical Support (CReDITSS), Level 4 West (Public Health), HMRI Building, NSW 2305 New Lambton Heights, Australia

**Keywords:** Gender equality, Women's rights, Maternal mortality, Empowerment, Structural equation modelling, Human rights

## Abstract

**Background:**

While a reduction in the global maternal mortality ratio (MMR) has slowed, newer strategies are needed to achieve an ongoing and sustainable reduction of the MMR. Previous studies have investigated the association between health system-related factors such as wealth inequalities, healthcare access and use on maternal mortality. However, a women’s rights-based approach to address MMR has not been studied, excluding the health system-related factors. This study aimed to analyse the association between gender equality and MMR globally.

**Methods:**

Using structural equation modelling (SEM), secondary and open access data from the United Nations and other international agencies from 193 countries were analysed using structural equation modelling (SEM). Gender-sensitive variables that represented the theoretical, conceptual framework of the study were selected. The association between latent variable gender equality and the outcome, MMR, was examined in the SEM. A second SEM model (*n* = 158) was designed to include two variables related to gender-based violence.

**Findings:**

The latent variable, gender equality, was negatively associated with MMR (*p* < 0‧001, Z = –6‧96, 95% CI: − 6508.98 to − 3141.89 for Model 1 and p < 0‧001, Z = –7‧23, 95% CI: − 6045.356 to − 3467.515 for Model 2).

**Interpretation:**

Gender equality was significantly associated with maternal mortality. Investing in higher education for women, improving their paid employment opportunities, increasing participation in leadership roles and politics, reducing intimate partner violence (IPV) and ending child marriage can significantly reduce maternal mortality.

**Supplementary Information:**

The online version contains supplementary material available at 10.1186/s12884-022-05225-6.

## Background

The premature death of a mother devastates the whole family. Beyond immense personal suffering to the family and loved ones, the trauma of a mother’s death can affect children throughout life. Maternal death negatively affects neonatal health, child survival, family functioning, child education, and the family’s socioeconomic status [1, 2]. These effects can have a multi-sectoral and intergenerational impact [1]. Maternal death is also multidimensional, and it can adversely impact the physical and mental health of family members throughout life [2]. The 2016 World Development Report suggests that approximately 830 women die daily due to complications in pregnancy and childbirth [3]. The maternal mortality ratio (MMR) ranged from 11 per 100,000 live births in high-income countries to 474 per 100,000 in low-income countries in 2016 [3]. Most maternal deaths are due to entirely preventable direct or indirect causes [4]. In published studies from 2003 to 2012, about 73% of maternal deaths were attributed to direct causes such as haemorrhage, sepsis and hypertensive disorders, while 27.5% of those deaths were due to indirect causes, like pre-existing medical disorders and HIV/AIDS [4]. However, maternal deaths due to abortion, obstructed labour, HIV/AIDs, and indirect causes were often misclassified and underreported, and therefore the exact number of maternal deaths might be larger than the estimated figs [4]. Under-reporting also occurs due to misattribution, misinformation, and complex personal and political reasons [4]. Indirect causes of maternal death are more complex than the known direct causes and, consequently, remain poorly addressed in global policies. Non-clinical interventions, such as improved governance and increased gender parity and women’s education, form a large part of addressing indirect causes of maternal deaths and have been highlighted in the 2014 World Health Organization report [5].

For the past several decades, the management of maternal mortality has been chiefly a risk-based approach [6]. Maternal risks have been treated using medicines and other clinical interventions. Essential interventions are directly influenced by women’s autonomy and rights, influenced by partner, family, community, culture, and religion [7, 8]. Despite its strength in reducing maternal mortality, contraception use largely depends on the autonomy i.e. the decision-making capacity and interpersonal relationships of a woman and her partner [7]. Joint household decision-making between the woman and their partners has positive associations with lower fertility, longer birth intervals and lower rates of unintended pregnancies [9]. Despite known effectiveness of modern contraceptives, an estimated 214 million women who wish to avert pregnancy do not use contraception due to factors closely related to a lack of reproductive and sexual autonomy, such as the experience of partner violence [9, 10]. In 2017, the unmet need of contraception low income countries was 24.52% and low and middle-income countries was 14.63%, which has not changed much since 2000 [11]. Reduced contraception use increases the risk of unintended pregnancies (mistimed or unwanted), abortions, and increased risk to adverse pregnancy outcomes [12]. Despite these known facts, access to modern contraception and abortion services remains challenging even in high income countries. Abortion laws are diverse, and huge variations can exist within countries [13]. Moreover, in countries where abortion is legal, decriminalised or permitted for psychological reasons, abortion services may not be readily available, accessible, or affordable [13]. It is evident that we need to look at unmet need of contraception, maternal mortality or other women’s health issues through the lens of gender equity.

Indicators of gender equality, such as women’s decision-making capacity and attitudes towards gender-based violence (GBV) in the African region, have shown a strong association with positive maternal health outcomes such as low body mass index and facility delivery [14]. The socio-political context of the women’s rights approach toward maternal health has been neglected for a long time despite successful strides in awareness and advocacy to improve gender equality. Therefore, improving maternal health needs a clear, multi-systemic women’s rights-based approach rather than solely concentrating on disease-based strategies like essential clinical interventions or family planning. These arguments also suggest that equality could be the most crucial marker that drives real and sustainable change. In order to examine gender equality, there is a need to be able to measure its concept accurately. Addressing this vital gap in literature can help in designing stronger policies.

Policies to reduce the persistent disadvantage suffered by women, such as inequality in education, lack of economic opportunities, and overall inequality that affects the development of a country, should be at the forefront of strategies to improve maternal health [15]. However, designing such policies requires extensive research and data that accurately define and measure gender equality. High-quality research on the effects of gender equality on sexual and reproductive health is limited [16]. What factors indicate gender equality and what can adequately be measured has been extensively debated and remains unclear. The World Health Organization (WHO) defines gender equality as the *‘equal chances or opportunities for groups of women and men to access and control social, economic and political resources, including protection under the law’* [17]. However, Lutwyche highlighted that “*the variables used to monitor the progress of gender equality were reductionist variables that fundamentally obscure the development of reality*” [18]. Reductionism drives much of the maternal mortality literature, avoiding the underlying, deep-rooted socio-political issues that affect women’s rights. GBV, a significant marker of gender equality, is a crucial omission in most gender equality literature.

Several issues highlight why women’s rights matter. The proportion of time spent undertaking unpaid care and domestic work by women is 2.6 times more compared to men globally, [19] making them more vulnerable to disadvantage and abuse. Women are under-represented in business ownership, leadership positions, and politics [19], thereby reducing their power to negotiate policies related to gender equality. Governments have been unsuccessful in providing essential protection against discrimination and violence due to weak laws on gender equality. Seventeen out of the 20 countries with the lowest female-employment-population ratios do not have nondiscrimination laws [19]. More than 45 countries do not have legislation on domestic violence or sexual harassment, and in 37 countries, rape perpetrators are exempted from prosecution if they marry the victim [20]. These unacceptable scenarios urge researchers to discover newer perspectives in analysing gender equality markers and substantiate their effect on maternal mortality. However, markers of gender equality, such as women’s education, employment, leadership roles, GBV and maternal health, have rarely been analysed concurrently. In another cross-sectional study, the indicators of gender equality were found to have a significant impact on under-five child mortality [21]. A systematic review has shown a concurrent effect of gender equality on child mortality; however, there are several knowledge gaps in the literature on gender equality and its impact on maternal health [22]. The review also indicated that a women’s rights-based approach is needed to improve maternal health globally [22]. Therefore, this study aimed to estimate the effect of gender equality markers on the reduction in maternal mortality for 193 countries.

## Methods

### Conceptual framework

**Fig. 1:**
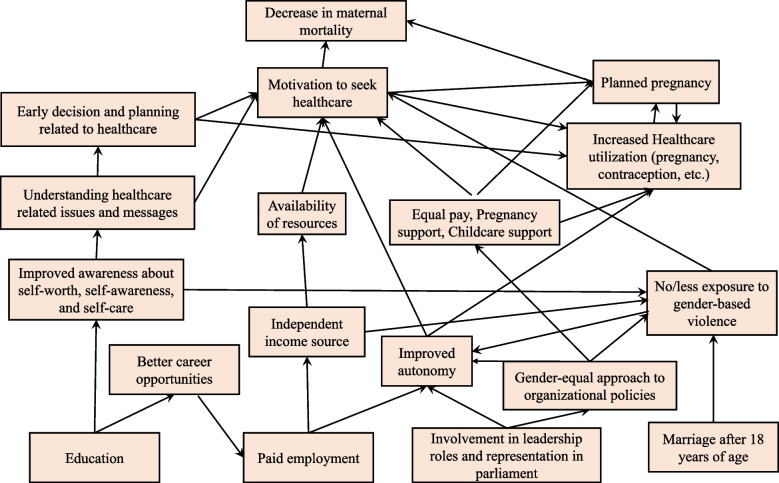
Hypothesised conceptual framework of the relationships between indicators of gender equality and maternal mortality

The hypothesised conceptual framework provides an overview of micro- to macro-level changes that can occur if different factors of gender equality are addressed in a policy. Education, paid employment, participation in leadership and parliament, reduction of GBV and child marriage can help to improve autonomy and, thereby, the motivation to use healthcare services and contraception [21, 22]. education can increase awareness about self-care, self-worth and self-awareness and help women reciprocate proactively to healthcare-related issues and messages [23]. As a result, women can become inclined to take early healthcare decisions, [23] a crucial factor for detecting complications in pregnancy. Education can also improve the opportunity for women to participate in paid employment and improve their career path compared to women who had no educational opportunity. Paid work enables empowerment and autonomy [24]. Moreover, financial autonomy is a vital aspect necessary for supporting the healthcare needs of women and their children [7, 24]. Participation of women in leadership and parliament helps in bringing attention to organisational and policy-level changes in favour of gender equality and reproductive health [25, 26]. Factors such as equal pay, parental leaves, childcare support and equal opportunities for women can help to enhance further women’s autonomy in both general and reproductive healthcare. The absence of violence and child marriage can improve autonomy [10, 27] and can provide an opportunity to fully participate and grow in all other domains of gender equality, such as education, paid employment and participation in leadership and parliament [21]. As a result of all these factors, healthcare service utilisation and contraception use can improve, resulting in a reduction in maternal mortality [22]. Figure 1 below was constructed using the existing literature and discussion with content experts regarding the directional relationships among variables that show the study’s conceptual framework.

### Study design

This study was a population-level analysis of open-source, secondary data between 2000 to 2017. The unit of analysis comprised aggregated summary statistics of relevant variables for each country. We have applied an identical research methodology reported in Bagade et al.(2022) [21] Free and open access data was retrieved from January 2018 to October 2018 from seven international organisations for 512 gender-sensitive variables representing 193 countries, free for public use under the Creative Commons Attribution 4‧0 (CC-BY 4‧0) international license [28–34]. Except for the *Maternal Mortality Rate (MMR)* variable, all other variables are the same as in the previous study [21]. Data cleaning and statistical analysis were performed using *MS-Excel*® and *Stata 15*®. Table 1 summarises the details of the variables used for the current study. Justification and description of backfilling method for data completeness are mentioned in Bagade et al.’s publication [21]. Multicollinearity was tested and found to be acceptable. Refer to Bagade et al. (2022) for further details of the study methods, handling of missing data, [21] and to the supplementary materials of this study for the detailed list of data sources, variables and their definitions.

**Table 1:** Details of all variables used for analysis to backfill missing data

No.	Variables^a^	Missing data (*n* = 193) (%)	Variable name used in SEM^b^	Missing data after backfilling (%)
1	Literacy rate	21.76	NA	
2	Main variable: Primary education attainment	29.53	Primary education attainment	2.07
	Proxy variables: Secondary enrolment—gross	5.18		
	Secondary enrolment—net	11.92		
	Progression to secondary school	16.58		
3	Main variable: Post-secondary education attainment	25.91	Secondary education attainment	6.22
	Proxy variables: Upper secondary education attainment	25.39		
	Lower secondary education completion	25.91		
	Tertiary school enrolment—gross	10.36		
	Education attainment—short tertiary	25.39		
4	Main variable: Education attainment—short tertiary	25.39	Tertiary education attainment	23.83
	Proxy variables: Education attainment—bachelor’s	49.74		
	Education attainment—master’s	54.40		
	Education attainment—doctoral	62.18		
5	Main variable: Firms with female ownership	25.39	Leadership participation	12.44
	Proxy variables: Households with a female head	62.69		
	Firms with a female in senior or middle management	50.26		
	Firms with females at top management	34.20		
6	Main variable: Child marriage	41.28	Child marriage	22.15
	Proxy variables: Married before 18 years of age	36.27		
	Married before 15 years of age	36.27		
7	Wage and salaried workers	8.29	NA	8.29
8	Female employers	8.29	NA	8.29
9	Vulnerable employment	8.29	NA	8.29
10	Female representation in parliament	0.52	NA	0.52
11	Intimate partner violence	44.04	NA	44.04
12	Maternal mortality rate	6.74	NA	6.74

^a^ Variables used to backfill missing data; ^b^ The variable name used in the structural equation model (SEM) after backfilling data from proxy variables; NA = Not applicable

### Statistical analysis

We designed two Structural Equation Models. Model 1 included nine variables, namely, literacy rate, primary education attainment, secondary education attainment, tertiary education attainment, waged and salaried workers, female employers, female share in leadership roles and their representation in parliament for 193 countries. Model 2 was a subgroup analysis that included two extra variables: IPV and child marriage for 158 nations. Thirty-seven countries with both missing variables were excluded in the Model 2 analysis.

**Fig. 2:**
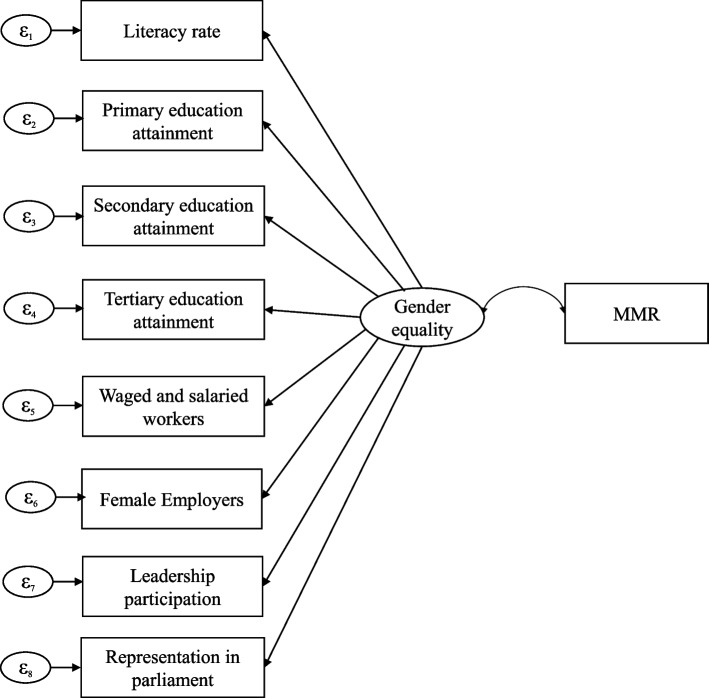
SEM Model 1

**Fig. 3:**
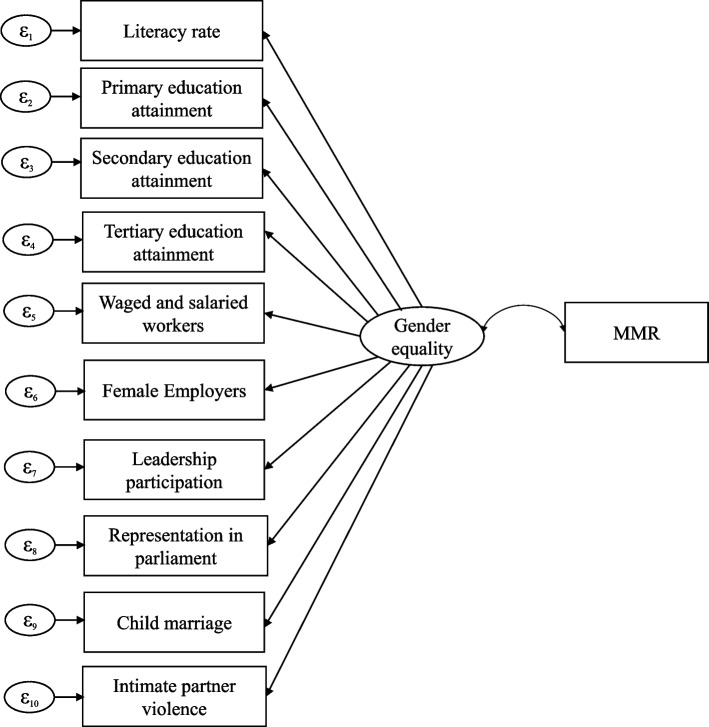
SEM Model 2 (subgroup analysis)

Gender equality was specified as a *latent* variable with paths to the *observed* variables. The outcome variable was MMR, and a covariance arrow indicated a correlation between latent (gender equality) and outcome (MMR) variables. Similar to our previously published study by Bagade et al., SEM estimates were analysed using *maximum likelihood with missing values* option and *robust variance estimation* (Huber/Whites/sandwich) to account for the non-normality and heteroscedasticity present in the variable distributions [21, 35–37]. The model estimates were assessed for the goodness of fit using a range of metrics, and statistical significance was indicated by *p* < 0‧05. The SEM diagrams for both models are mentioned in Figs. 2 and 3 below.

## Results

Convergence was achieved in both the SEMs. The model estimates demonstrated that the indicators of gender equality (primary, secondary and tertiary education attainment, waged and salaried employment, female employers and women’s representation in leadership roles) were statistically significantly associated with MMR. As gender equality increased, MMR decreased (Z score = − 6‧95, CI: − 5608.979 to − 3141.895, *p* < 0‧001, *n* = 193 for Model 1, and Z = –7‧23, CI: − 6045.356 to − 3467.515, *p* < 0‧001, *n* = 158 for Model 2). Additionally in Model 2, child marriage and IPV were found to be negatively associated with gender equality. In both models, the coefficients of the hypothesised associations were an excellent fit for the observed data. The following tables show the different components of the SEM results. Tables 2 and 3 show the variable summaries, where the means of variables are reported as percentage per population. Tables 4, 5, 6 and 7 have the estimation results and model variance, whereas Table 8 contains goodness of fit indicators, namely, Goodness of fit index, Akaike Information Criterion and the Bayesian Information Criterion of both the models.

**Table 2:** SEM Model 1 variables summary statistics

	Variable name	n	Missing data (%)	Mean (%)	Std dev.	Min.	Max.
1	Literacy rate	151	21.76	77.97	24.90	13.96	99.99
2	Primary school education attainment	189	2.07	74.97	26.46	2.56	118.69
3	Secondary school education attainment	181	6.22	22.15	18.90	0	104.67
4	Tertiary education attainment	147	23.83	18.39	15.18	0	63.69
5	Waged and salaried workers	177	8.29	56.50	31.64	1.03	99.61
6	Female employers	177	8.29	1.81	1.45	0.04	10.77
7	Women in leadership roles	169	12.44	34.96	15.96	2.2	86.8
8	Women in parliament	192	0.52	21.27	11.77	0	61.3
9	Maternal mortality ratio	180	6.74	171.14	233.53	3	1360

**Table 3:** SEM Model 2 (subgroup analysis) summary statistics

	Variable name	n	Missing data (%)	Mean (%)	Std dev.	Min.	Max.
1	Literacy rate	132	16.46	75.67	25.77	13.96	99.98
2	Primary school education attainment	155	1.90	71.49	27.58	2.56	118.69
3	Secondary school education attainment	149	5.70	19.14	16.78	0	75.15
4	Tertiary education attainment	123	22.15	16.80	14.56	0	58.57
5	Waged and salaried workers	152	3.80	52.16	31.37	1.03	99.61
6	Female employers	152	3.80	1.815	1.51	0.04	10.77
7	Women in leadership roles	148	6.33	34.31	15.65	2.2	86.8
8	Women in parliament	158	0	21.86	12.195	0	61.3
9	Intimate partner violence prevalence	107	32.28	28.35	13.74	6.1	67.6
10	Child marriage	123	22.15	23.49	15.06	1.6	76.3
11	Maternal mortality ratio	154	2.53	194.70	244.46	3	1360

**Table 4:** SEM Model 1 estimation results

Variable	Coefficient	Standard error	Z-score	*p*-value	95% Confidence interval	
Literacy rate	1 (constrained)					
Primary school education attainment	1.03	0.08	13.86	< 0.001	0.89	1.18
Secondary school education attainment	0.62	0.063	9.95	< 0.001	0.50	0.75
Tertiary education attainment	0.51	0.05	9.52	< 0.001	0.41	0.62
Waged and salaried workers	1.22	0.08	16.17	< 0.001	1.07	1.37
Female employers	0.02	0.003	4.01	< 0.001	0.008	0.02
Women in leadership roles	0.15	0.05	3.12	0.002	0.05	0.24
Women in parliament	0.07	0.04	1.95	0.052	−0.0005	0.15
Mean maternal mortality ratio	163.06	16.62	9.81	< 0.001	130.48	195.64
cov (mmr, gender_equality)	− 4375.437	629.3697	−6.95	< 0.001	−5608.979	−3141.895

**Table 5:** Model 1 variance

var(e.literacy_1)	Coefficient	Standard Error	95% Confidence interval	
	128.5385	26.30413	86.06829	191.9655
var(e.prim_edu_1)	164.553	25.04261	122.114	221.7413
var(e.sec_edu_1)	163.5088	36.60976	105.4281	253.5864
var(e.tertiary_edu)	107.9031	16.65968	79.72835	146.0343
var(e.wage_salaried1)	228.0603	33.22057	171.4189	303.4175
var(e.employers1)	1.971189	0.5464386	1.144892	3.393846
var(e.leadership_1)	242.3653	27.20359	194.5045	302.0029
var(e.parliament1)	135.1825	14.13872	110.1269	165.9387
var (mmr_modelled_est1)	53,678.92	9851.989	37,460.89	76,918.27
var (gender_equality)	505.7647	73.01678	381.1196	671.1749

**Table 6:** SEM Model 2 (subgroup analysis) estimation results

Variable	Coefficient	Standard Error	Z-score	*p*-value	95% Confidence interval	
Literacy rate	1 (constrained)					
Primary school education attainment	1.033	0.07	14.60	< 0.001	0.89	1.17
Secondary school education attainment	0.5	0.06	9.71	< 0.001	0.44	0.66
Tertiary education attainment	0.47	0.05	9.33	< 0.001	0.37	0.57
Waged and salaried workers	1.19	0.07	16.02	< 0.001	1.04	1.33
Female employers	0.02	0.004	4.23	< 0.001	0.009	0.03
Women in leadership roles	0.12	0.05	2.69	0.007	0.03	0.22
Women in parliament	0.099	0.04	2.50	0.012	0.02	0.17
Intimate partner violence	−0.19	0.05	−3.61	< 0.001	−0.29	−0.09
Child marriage	−0.52	0.05	−10.74	< 0.001	−0.62	− 0.43
Mean Maternal mortality ratio	192.05	19.40	9.90	< 0.001	154.02	230.08
cov (mmr, gender_equality)	− 4756.436	657.6246	−7.23	< 0.001	−6045.356	−3467.515

**Table 7:** Model 2 variance

var(e.literacy_1)	Coefficient	Standard error	95% Confidence interval	
	140.5218	26.20233	97.50455	202.5175
var(e.prim_edu_1)	178.813	28.14916	131.3412	243.443
var(e.sec_edu_1)	118.0998	20.83857	83.57099	166.8947
var(e.tertiary_edu)	95.56875	16.20038	68.55263	133.2317
var(e.wage_salaried1)	197.7267	30.00076	146.8635	266.2054
var(e.employers1)	2.097429	0.6255123	1.169056	3.763046
var(e.leadership_1)	234.797	29.97203	182.8252	301.543
var(e.parliament1)|	142.5243	16.21567	114.0364	178.1288
var(e.child_marriage_0)|	100.9026	15.83051	74.19221	137.2292
var(e.IPV)	167.3436	26.0699	123.3114	227.0988
var (mmr_modelled_est1)	59,072.96	10,724.7	41,385.96	84,318.8
var (Gender_equality)|	545.036	75.48362	415.4702	715.0075

**Table 8:** Goodness of fit comparison Model 1 v. Model 2 subgroup analysis

Factor	Model 1	Model 2 (subgroup analysis)
n	193	158
Goodness of Fit Index	0.929	0.939
Akaike information criterion	13,002.02	12,776.05
Bayes information criterion	13,090.12	12,877.12

## Discussion

Improvement in gender equality significantly reduced maternal mortality. Attainment of education at primary, secondary and tertiary levels, waged and salaried employment, female employers, participation in leadership roles and representation in parliament improved gender equality, whereas IPV and child marriage adversely affected gender equality. The findings of the study are similar to the previous research by the same researchers that analysed the effect of gender equality on under-five child mortality [21]. However, this is the first time that a conceptual framework of gender equality has been proposed in maternal mortality and analysed using data from 193 countries. The attempt to provide a starting point for using a women’s rights approach towards policy changes in health is novel, bold and complex, but not without some limitations.

We used a gendered lens and women’s rights-based approach toward maternal health, but the study was not without limitations. Causes of maternal death are multi-systemic, ranging from the health system to geographical, economic and socio-political issues. Numerous confounders and covariates that affect maternal health may have affected the results of the study. Secondly, this study used summary statistics, and data reporting remained weak, primarily where GBV was concerned. However, the statistical method, SEM, and the optimisation methods used in our study assisted with analysing a small sample with missing observations. Causal relationships cannot be established due to challenges in data reporting by international agencies that mainly depend on the quality of data reported by different countries. Although, this challenge highlights the need for better reporting of variables identified in our study. We have recognized the specific variables that were associated with gender equality. The literature is scarce on studies that include GBV as a core component of gender equality; therefore, the study is a vital contribution to the literature. The variables used for both statistical models can be used as a benchmark for collecting and reporting data on gender equality. The crucial findings of our study will significantly contribute to the existing literature and pave the way for future researchers to use a women’s rights approach toward development policies.

Measuring gender equality beyond education-only indicators will help develop a structured approach toward policies. However, female education is an important marker of gender equality, so much so that the MDG goal on gender equality prioritised improving equality in education [38]. Female literacy and level of education have a strong relationship with maternal mortality globally [23]. Maternal mortality is low in countries where women’s status is high, especially when women are educated [39]. Education improves health awareness and response to health-related messages, and a higher education level attainment improves autonomy and motivation to seek healthcare. Importantly, contraception use has been found to increase among women with higher education [40]. Educated women have a better capacity to analyse the contraceptive choices that better suit their needs [41]. More extended duration of female education can enhance chances of paid employment, control fertility, prevent child marriage, delay fertility, and thereby reduce the risk of maternal deaths [23, 40]. Strategic policies to improve funding for the higher education of women can yield better health outcomes than focusing only on literacy rate or primary education, which, according to our study results, should be considered necessary but insufficient conditions for gender equality.

Paid employment relates to women’s financial autonomy [42]. Financial autonomy involving several dimensions, such as achieving economic independence, having control over one’s monetary affairs and exercising agency concerning household and personal spending, is an essential factor in couple relationships [43]. Financially independent women were more likely to have better health outcomes than men in their later lives [42]. Waged and salaried employment status and self-employed women who are employers are significantly associated with improving gender equality.

In this study, the proportion of women represented in the lower or upper house of parliament showed a strong association with gender equality and a reduction in maternal deaths. Parliamentarians play a vital role in connecting population policies and social development, as the gender of legislators influences their policy priorities [44]. Thus, women’s empowerment in the political landscape has the potential to change society [44]. Female parliamentarians are strong advocates of child health and women’s rights policies [44]. Democracy is more robust only when it is fully inclusive of the population it represents, and women representing half the global population should have equal status and participatory rights in the parliament of a country [26]. Researchers have shown that female legislators have supported and advocated health, education, and gender equality policies [25]. Female parliamentarians emphasise policies that prioritise not only women but children, ethnic and racial minorities, and marginalised populations [25]. Decisions made by female politicians are helpful in peace-building negotiations and post-conflict recovery [25]. Improved public provision of antenatal services and childhood health services was evident in some states of India, where there were more female parliamentarians [45]. A 10% increase in female representation in politics can decrease neonatal mortality by 2.1% [45].

Female leadership representation is another crucial marker of gender equality [46]. Women in leadership positions indicate a society that has reduced barriers that discriminate against women because of their gender [46]. Equal participation in leadership positions can discourage workplace discrimination and promote an ideal, highly productive workforce [46]. Our study revealed a strong association between the percentage of female leaders and maternal mortality. We could not find studies that have analysed this relationship, so more studies are needed to find the possible mechanisms of how leadership roles improve maternal health.

GBV has become a global issue of epidemic proportion, affecting millions of people’s physical and emotional well-being over the life course. Despite international efforts, standard classification and enough data for variables measuring GBV have remained a challenge. In this study, the prevalence of child marriage and IPV was negatively associated with gender equality. IPV negatively affects women’s reproductive health outcomes [10]. GBV and other indicators of gender equality are significantly associated with women’s autonomy. Policies to strengthen healthcare interventions, clinical care, and family planning are undermined by women’s fundamental rights and autonomy issues [47]. In low and middle-income countries, decision-making regarding access and use of skilled maternal healthcare services is influenced by the values and opinions of husbands, mothers-in-law, traditional birth attendants, religious leaders, and other family and community members rather than the individual women [48]. Women’s lack of autonomy directly affects maternal health outcomes such as antenatal care utilization, skilled attendance at birth, and contraception use [47]. Therefore, through policies addressing gender equality, women’s empowerment is the only way to improve their autonomy and reproductive health-seeking behaviour [48].

More than 50 years since the Alma-Ata Declaration, which promised health for all by 2000, preventable maternal deaths are still high. Treaties such as the Convention on the Elimination of all Forms of Discrimination Against Women, the Beijing Declaration Plan of Action, and the International Conference on Population and Development’s Plan of Action strategised the movement toward gender equality [49]. However, unanticipated conflicts, natural disasters, epidemics and war during the past two decades have slowed the progress in strengthening women’s rights. Further, the economic downturn due to the COVID-19 pandemic is predicted to impact gender equality significantly [50]. While arguments for defining gender equality were justified, researchers have struggled to find a common consensus of alternatives to address the vast research gaps. Our study has tried to fill a significant gap in the literature and has been a momentous step in strategic thinking about the effects of gender equality on maternal health. We now have the exact list of variables that need to be continuously monitored and reported to analyse progress. However, we have also identified several gaps in the literature that will require numerous more studies that keep women’s rights at the centre of women’s health, rather than just a disease-centred approach.

## Conclusion

Reforms to address women’s rights are the most urgent priority to expedite better health for future generations. Gender equality intersects all other global developmental goals, and its importance is beyond just women’s well-being. The multi-sectoral policy emphasis on gender equality can instigate change that can directly reduce overall inequality worldwide. Increasing funding for higher education, improving opportunities for paid employment, increasing women’s representation in leadership and politics, and strengthening laws and policies to eliminate child marriages and IPV is the key recommendations concluded from the study. To achieve this, we need united global leadership, consistent advocacy, and most of all, a political will to focus on women’s rights at the national level.

## Supplementary Information

**Additional file 1.**

## Data Availability

The authors have provided the links to the open-source data available on the external organisation’s websites in the supplementary material. The data is publicly accessible.

## References

[1] Miller S, Belizán JM (2015). The true cost of maternal death: individual tragedy impacts family, community and nations. Reprod Health.

[2] Zhou H, Zhang L, Ye F, Wang H-J, Huntington D, Huang Y, Wang A, Liu S, Wang Y (2016). The effect of maternal death on the health of the husband and children in a rural area of China: a prospective cohort study. PLoS One.

[3] TheWorldBank (2016). 2016 World Development Indicators.

[4] Say L, Chou D, Gemmill A, Tunçalp Ö, Moller A-B, Daniels J, Gülmezoglu AM, Temmerman M, Alkema L (2014). Global causes of maternal death: a WHO systematic analysis. Lancet Glob Health.

[5] WHO (2014). Sucess factors for women's and children's health: policy and programme highlights from 10 fast-track countries.

[6] Winikoff B (1995). Is the risk approach effective in maternal care?. Safe Mother.

[7] Senarath U, Gunawardena NS (2009). Women's autonomy in decision making for health Care in South Asia. Asia Pac J Public Health.

[8] Jejeebhoy SJ, Sathar ZA (2001). Women's autonomy in India and Pakistan: the influence of religion and region. Popul Dev Rev.

[9] Upadhyay UD, Gipson JD, Withers M, Lewis S, Ciaraldi EJ, Fraser A, Huchko MJ, Prata N (2014). Women's empowerment and fertility: a review of the literature. Soc Sci Med.

[10] Sarkar NN (2008). The impact of intimate partner violence on women's reproductive health and pregnancy outcome. J Obstet Gynaecol.

[11] World Bank. World development indicators 2017. The World Bank; 2017.

[12] Ahmed S, Li Q, Liu L, Tsui AO (2012). Maternal deaths averted by contraceptive use: an analysis of 172 countries. Lancet.

[13] Johnson BR, Mishra V, Lavelanet AF, Khosla R, Ganatra B (2017). A global database of abortion laws, policies, health standards and guidelines. Bull World Health Organ.

[14] Singh K, Bloom S, Brodish P (2015). Gender equality as a means to improve maternal and child health in Africa. Health Care Women Int.

[15] Grown C, Gupta GR, Pande R (2005). Taking action to improve women's health through gender equality and women's empowerment. Lancet.

[16] Hartmann M, Khosla R, Krishnan S, George A, Gruskin S, Amin A (2016). How are gender equality and human rights interventions included in sexual and reproductive health programmes and policies: a systematic review of existing research foci and gaps. PLoS One.

[17] Glossary of terms and tools [http://www.who.int/gender-equity-rights/knowledge/glossary/en/].

[18] Lutwyche W (2012). Framing gender inequality: millennium development goal 3 and the Post-2015 agenda. ANU Undergrad Res J.

[19] WorldBank (2018). Atlas of sustainable development goals 2018 from world development indicators.

[20] Women U (2018). Summary: turning promises into action: gender equality in the 2030 agenda for sustainable development.

[21] Bagade T, Chojenta C, Harris M, Oldmeadow C, Loxton D (2022). A Women's rights-based approach to reducing child mortality: Data from 193 countries show that gender equality does affect under-five child mortality. Matern Child Health J.

[22] Bagade T, Chojenta C, Harris ML, Nepal S, Loxton D (2019). Does gender equality and availability of contraception influence maternal and child mortality? A systematic review. BMJ Sex Reprod Health.

[23] McAlister C, Baskett TF (2006). Female education and maternal mortality: a worldwide survey. J Obstet Gynaecol Can.

[24] Taguchi N, Kawabata M, Maekawa M, Maruo T, Aditiawarman DL (2003). Influence of socio-economic background and antenatal care programmes on maternal mortality in Surabaya, Indonesia. Trop Med Int Health.

[25] Markham S (2012). Strengthening Women's roles in parliaments. Parliam Aff.

[26] IPU (2008). Equality in politics: a survey of men and women in parliaments.

[27] Godha D, Hotchkiss DR, Gage AJ (2013). Association between child marriage and reproductive health outcomes and service utilization: a multi-country study from South Asia. J Adolesc Health.

[28] Attribution 4.0 International (CC BY 4.0) [https://creativecommons.org/licenses/by/4.0/ ].

[29] ILOSTAT-The leading source of labour statistics - Bulk download facility [https://ilostat.ilo.org/data/bulk/].

[30] Global data on national parliaments [https://data.ipu.org/search].

[31] Bulk Data Download Service [https://apiportal.uis.unesco.org/bdds].

[32] Human Development Data (1990-2018) [http://hdr.undp.org/en/data].

[33] UN datamart Explorer [http://data.un.org/Explorer.aspx].

[34] Search and Share Development data [https://datacatalog.worldbank.org/].

[35] Dong Y (2013). Peng C-YJ: principled missing data methods for researchers. SpringerPlus.

[36] Cheng EWL (2001). SEM being more effective than multiple regression in parsimonious model testing for management development research. J Manag Dev.

[37] Savalei V (2014). Understanding robust corrections in structural equation Modeling. Struct Equ Model Multidiscip J.

[38] Unterhalter E (2005). Global inequality, capabilities, social justice: the millennium development goal for gender equality in education. Int J Educ Dev.

[39] Filippi V, Ronsmans C, Campbell OMR, Graham WJ, Mills A, Borghi J, Koblinsky M, Osrin D (2006). Maternal health in poor countries: the broader context and a call for action. Lancet.

[40] Koch E, Calhoun B, Aracena P, Gatica S, Bravo M (2014). Women's education level, contraceptive use and maternal mortality estimates. Public Health.

[41] Snopkowski K, Towner MC, Shenk MK, Colleran H (2016). Pathways from education to fertility decline: a multi-site comparative study. Philos Trans R Soc Lond Ser B Biol Sci.

[42] Roy K, Chaudhuri A (2008). Influence of socioeconomic status, wealth and financial empowerment on gender differences in health and healthcare utilization in later life: evidence from India. Soc Sci Med.

[43] Bennett F, Sung S (2013). Dimensions of financial autonomy in low−/moderate-income couples from a gender perspective and implications for welfare reform. J Soc Policy.

[44] Moccia P, Anthony D (2006). The state of the world's children 2007: women and children: the double dividend of gender equality.

[45] Bhalotra S, Clots-Figueras I (2014). Health and the political Agency of Women. Am Econ J Econ Pol.

[46] Miller AR (2017). Women and leadership. Oxford Handbook on Women and the Economy.

[47] Pratley P (2016). Associations between quantitative measures of women's empowerment and access to care and health status for mothers and their children: a systematic review of evidence from the developing world. Soc Sci Med.

[48] Ganle JK, Obeng B, Segbefia AY, Mwinyuri V, Yeboah JY, Baatiema L (2015). How intra-familial decision-making affects women’s access to, and use of maternal healthcare services in Ghana: a qualitative study. BMC Pregnancy Childbirth.

[49] CEDAW and Human rights - Frequently asked questions (FAQ) about CEDAW [http://asiapacific.unwomen.org/en/focus-areas/cedaw-human-rights/faq#whatstateparties].

[50] Alon TM, Doepke M, Olmstead-Rumsey J, Tertilt M (2020). The impact of COVID-19 on gender equality.

