# Supplementary material for: The human right to safely give birth: data from 193 countries show that gender equality does affect maternal mortality
Source: BMC Pregnancy Childbirth. 2022 Nov 24;22:874. doi: 10.1186/s12884-022-05225-6 (PMC9685845; doi:10.1186/s12884-022-05225-6)
Supplement: Supplementary file 1 — Additional file 1. [file 12884_2022_5225_MOESM1_ESM.docx]

**Supplementary material**

1. **Indicator name list**
2. **Data dictionary for the indicators selected for final analysis**
3. **Indicator Name list**
4. Death rate, crude (per 1,000 people)
5. Decision maker about a woman's own health care: mainly husband (% of women age 15-49)
6. Decision maker about a woman's own health care: mainly wife  (% of women age 15-49)
7. Decision maker about a woman's own health care: other (% of women age 15-49)
8. Decision maker about a woman's own health care: someone else (% of women age 15-49)
9. Decision maker about a woman's own health care: wife and husband jointly (% of women age 15-49)
10. Decision maker about a woman's visits to her family or relatives: mainly husband (% of women age 15-49)
11. Decision maker about a woman's visits to her family or relatives: mainly wife (% of women age 15-49)
12. Decision maker about a woman's visits to her family or relatives: other (% of women age 15-49)
13. Decision maker about a woman's visits to her family or relatives: someone else (% of women age 15-49)
14. Decision maker about major household purchases: mainly husband (% of women age 15-49)
15. Decision maker about major household purchases: mainly wife (% of women age 15-49)
16. Decision maker about major household purchases: other (% of women age 15-49)
17. Decision maker about major household purchases: someone else (% of women age 15-49)
18. Decision maker about major household purchases: wife and husband jointly (% of women age 15-49)
19. Decision maker about Visits to her family or relatives: wife and husband jointly (% of women age 15-49)
20. Demand for family planning satisfied by any methods (% of married women with demand for family planning)
21. Demand for family planning satisfied by modern methods (% of married women with demand for family planning)
22. Educational attainment, at least Bachelor's or equivalent, population 25+, female (%) (cumulative)
23. Educational attainment, at least Bachelor's or equivalent, population 25+, male (%) (cumulative)
24. Educational attainment, at least completed lower secondary, population 25+, female (%) (cumulative)
25. Educational attainment, at least completed lower secondary, population 25+, male (%) (cumulative)
26. Educational attainment, at least completed post-secondary, population 25+, female (%) (cumulative)
27. Educational attainment, at least completed post-secondary, population 25+, male (%) (cumulative)
28. Educational attainment, at least completed primary, population 25+ years, female (%) (cumulative)
29. Educational attainment, at least completed primary, population 25+ years, male (%) (cumulative)
30. Educational attainment, at least completed short-cycle tertiary, population 25+, female (%) (cumulative)
31. Educational attainment, at least completed short-cycle tertiary, population 25+, male (%) (cumulative)
32. Educational attainment, at least completed upper secondary, population 25+, female (%) (cumulative)
33. Educational attainment, at least completed upper secondary, population 25+, male (%) (cumulative)
34. Educational attainment, at least Master's or equivalent, population 25+, female (%) (cumulative)
35. Educational attainment, at least Master's or equivalent, population 25+, male (%) (cumulative)
36. Educational attainment, Doctoral or equivalent, population 25+, female (%) (cumulative)
37. Educational attainment, Doctoral or equivalent, population 25+, male (%) (cumulative)
38. Employers, female (% of female employment) (modeled ILO estimate)
39. Employers, male (% of male employment) (modeled ILO estimate)
40. Employment in agriculture, female (% of female employment) (modeled ILO estimate)
41. Employment in agriculture, male (% of male employment) (modeled ILO estimate)
42. Employment in industry, female (% of female employment) (modeled ILO estimate)
43. Employment in industry, male (% of male employment) (modeled ILO estimate)
44. Employment in services, female (% of female employment) (modeled ILO estimate)
45. Employment in services, male (% of male employment) (modeled ILO estimate)
46. Employment to population ratio, 15+, female (%) (modeled ILO estimate)
47. Employment to population ratio, 15+, female (%) (national estimate)
48. Employment to population ratio, 15+, male (%) (modeled ILO estimate)
49. Employment to population ratio, 15+, male (%) (national estimate)
50. Employment to population ratio, 15+, total (%) (national estimate)
51. Employment to population ratio, ages 15-24, female (%) (modeled ILO estimate)
52. Employment to population ratio, ages 15-24, female (%) (national estimate)
53. Employment to population ratio, ages 15-24, male (%) (modeled ILO estimate)
54. Employment to population ratio, ages 15-24, male (%) (national estimate)
55. Employment to population ratio, ages 15-24, total (%) (national estimate)
56. Expected Years of School
57. Expected Years of School, Female
58. Expected Years of School, Male
59. Expected years of schooling, female
60. Expected years of schooling, male
61. Female genital mutilation prevalence (%)
62. Female genital mutilation prevalence (%): Q1 (lowest)
63. Female genital mutilation prevalence (%): Q2
64. Female genital mutilation prevalence (%): Q3
65. Female genital mutilation prevalence (%): Q4
66. Female genital mutilation prevalence (%): Q5 (highest)
67. Female headed households (% of households with a female head)
68. Female migrants (% of international migrant stock)
69. Female professional and technical workers (% of total)
70. Female share of employment in senior and middle management (%)
71. Fertility rate, total (births per woman)
72. Firms with female participation in ownership (% of firms)
73. Firms with female top manager (% of firms)
74. Fraction of Children Under 5 Not Stunted
75. Fraction of Children Under 5 Not Stunted, Female
76. Fraction of Children Under 5 Not Stunted, Male
77. GDP (current US$)
78. GDP growth (annual %)
79. GDP per capita (current US$)
80. GINI index (World Bank estimate)
81. GNI per capita, Atlas method (current US$)
82. GNI per capita, PPP (current international $)
83. GNI, Atlas method (current US$)
84. Government expenditure on education, total (% of GDP)
85. Government expenditure per student, primary (% of GDP per capita)
86. Government expenditure per student, secondary (% of GDP per capita)
87. Primary completion rate, female, based on completers
88. Primary completion rate, male, based on completers
89. Gross graduation ratio, tertiary, female (%)
90. Gross graduation ratio, tertiary, male (%)
91. Harmonized Test Scores
92. Harmonized Test Scores, Female
93. Harmonized Test Scores, Male
94. Households with water 30 minutes or longer away round trip (%)
95. Households with water less than 30 minutes away round trip (%)
96. Households with water on the premises (%)
97. Human Capital Index (HCI) (scale 0-1)
98. Human Capital Index (HCI), Female (scale 0-1)
99. Human Capital Index (HCI), Female, Lower Bound (scale 0-1)
100. Human Capital Index (HCI), Female, Upper Bound (scale 0-1)
101. Human Capital Index (HCI), Lower Bound (scale 0-1)
102. Human Capital Index (HCI), Male (scale 0-1)
103. Human Capital Index (HCI), Male, Lower Bound (scale 0-1)
104. Human Capital Index (HCI), Male, Upper Bound (scale 0-1)
105. Human Capital Index (HCI), Upper Bound (scale 0-1)
106. Immunization, DPT (% of children ages 12-23 months)
107. Immunization, measles (% of children ages 12-23 months)
108. Inflation, consumer prices (annual %)
109. Informal employment, female (% of total non-agricultural employment)
110. Informal employment, male (% of total non-agricultural employment)
111. Labor force participation rate for ages 15-24, female (%) (modeled ILO estimate)
112. Labor force participation rate for ages 15-24, female (%) (national estimate)
113. Labor force participation rate for ages 15-24, male (%) (modeled ILO estimate)
114. Labor force participation rate for ages 15-24, male (%) (national estimate)
115. Labor force participation rate, female (% of female population ages 15+) (modeled ILO estimate)
116. Labor force participation rate, female (% of female population ages 15+) (national estimate)
117. Labor force participation rate, male (% of male population ages 15+) (modeled ILO estimate)
118. Labor force participation rate, male (% of male population ages 15+) (national estimate)
119. Labor force with advanced education, female (% of female working-age population with advanced education)
120. Labor force with advanced education, male (% of male working-age population with advanced education)
121. Labor force with basic education, female (% of female working-age population with basic education)
122. Labor force with basic education, male (% of male working-age population with basic education)
123. Labor force with intermediate education, female (% of female working-age population with intermediate education)
124. Labor force with intermediate education, male (% of male working-age population with intermediate education)
125. Labor force, female
126. Labor force, female (% of total labor force)
127. Labor force, total
128. Law mandates equal remuneration for females and males for work of equal value (1=yes; 0=no)
129. Law mandates nondiscrimination based on gender in hiring (1=yes; 0=no)
130. Law mandates paid or unpaid maternity leave (1=yes; 0=no)
131. Law prohibits or invalidates child or early marriage (1=yes; 0=no)
132. Learning-Adjusted Years of School
133. Learning-Adjusted Years of School, Female
134. Learning-Adjusted Years of School, Male
135. Legislation exists on domestic violence (1=yes; 0=no)
136. Legislation explicitly criminalizes marital rape (1=yes; 0=no)
137. Legislation specifically addresses sexual harassment (1=yes; 0=no)
138. Life expectancy at age 60, female
139. Life expectancy at age 60, male
140. Life expectancy at birth, female (years)
141. Life expectancy at birth, male (years)
142. Literacy rate, adult female (% of females ages 15 and above)
143. Literacy rate, adult male (% of males ages 15 and above)
144. Literacy rate, youth (ages 15-24), gender parity index (GPI)
145. Literacy rate, youth female (% of females ages 15-24)
146. Literacy rate, youth male (% of males ages 15-24)
147. Location of cooking: inside the house (% of households)
148. Location of cooking: other places (% of households)
149. Location of cooking: outdoors (% of households)
150. Location of cooking: separate building (% of households)
151. Lower secondary completion rate, female (% of relevant age group)
152. Lower secondary completion rate, male (% of relevant age group)
153. Main cooking fuel: agricultural crop (% of households)
154. Main cooking fuel: charcoal (% of households)
155. Main cooking fuel: dung (% of households)
156. Main cooking fuel: electricity (% of households)
157. Main cooking fuel: LPG/natural gas/biogas (% of households)
158. Main cooking fuel: straw/shrubs/grass (% of households)
159. Main cooking fuel: wood (% of households)
160. Married men and married women have equal ownership rights to property (1=yes; 0=no)
161. Married women are required by law to obey their husbands (1=yes; 0=no)
162. Married women can obtain a national ID card in the same way as married men (1=yes; 0=no)
163. Maternal mortality ratio (modeled estimate, per 100,000 live births)
164. Maternity leave (days paid)
165. Maternal leave benefits (% of wages paid)
166. Men who do not own a house (% of men)
167. Men who do not own a house (% of men): Q1 (lowest)
168. Men who do not own a house (% of men): Q2
169. Men who do not own a house (% of men): Q3
170. Men who do not own a house (% of men): Q4
171. Men who do not own a house (% of men): Q5 (highest)
172. Men who do not own land (% of men)
173. Men who do not own land (% of men): Q1 (lowest)
174. Men who do not own land (% of men): Q2
175. Men who do not own land (% of men): Q3
176. Men who do not own land (% of men): Q4
177. Men who do not own land (% of men): Q5 (highest)
178. Men who own a house alone (% of men)
179. Men who own a house alone (% of men): Q1 (lowest)
180. Men who own a house alone (% of men): Q2
181. Men who own a house alone (% of men): Q3
182. Men who own a house alone (% of men): Q4
183. Men who own a house alone (% of men): Q5 (highest)
184. Men who own a house both alone and jointly (% of men)
185. Men who own a house both alone and jointly (% of men): Q1 (lowest)
186. Men who own a house both alone and jointly (% of men): Q2
187. Men who own a house both alone and jointly (% of men): Q3
188. Men who own a house both alone and jointly (% of men): Q4
189. Men who own a house both alone and jointly (% of men): Q5 (highest)
190. Men who own a house jointly (% of men)
191. Men who own a house jointly (% of men): Q1 (lowest)
192. Men who own a house jointly (% of men): Q2
193. Men who own a house jointly (% of men): Q3
194. Men who own a house jointly (% of men): Q4
195. Men who own a house jointly (% of men): Q5 (highest)
196. Men who own land alone (% of men)
197. Men who own land alone (% of men): Q1 (lowest)
198. Men who own land alone (% of men): Q2
199. Men who own land alone (% of men): Q3
200. Men who own land alone (% of men): Q4
201. Men who own land alone (% of men): Q5 (highest)
202. Men who own land both alone and jointly (% of men)
203. Men who own land both alone and jointly (% of men): Q1 (lowest)
204. Men who own land both alone and jointly (% of men): Q2
205. Men who own land both alone and jointly (% of men): Q3
206. Men who own land both alone and jointly (% of men): Q4
207. Men who own land both alone and jointly (% of men): Q5 (highest)
208. Men who own land jointly (% of men)
209. Men who own land jointly (% of men): Q1 (lowest)
210. Men who own land jointly (% of men): Q2
211. Men who own land jointly (% of men): Q3
212. Men who own land jointly (% of men): Q4
213. Men who own land jointly (% of men): Q5 (highest)
214. Mortality from CVD, cancer, diabetes or CRD between exact ages 30 and 70, female (%)
215. Mortality from CVD, cancer, diabetes or CRD between exact ages 30 and 70, male (%)
216. Mortality rate attributed to household and ambient air pollution, age-standardized, female (per 100,000 female population)
217. Mortality rate attributed to household and ambient air pollution, age-standardized, male (per 100,000 male population)
218. Mortality rate attributed to unintentional poisoning, female (per 100,000 female population)
219. Mortality rate attributed to unintentional poisoning, male (per 100,000 male population)
220. Mortality rate, infant, female (per 1,000 live births)
221. Mortality rate, infant, male (per 1,000 live births)
222. Mortality rate, under-5, female (per 1,000 live births)
223. Mortality rate, under-5, male (per 1,000 live births)
224. Mothers are guaranteed an equivalent position after maternity leave (1=yes; 0=no)
225. Net intake rate in grade 1, female (% of official school-age population)
226. Net intake rate in grade 1, male (% of official school-age population)
227. Nondiscrimination clause mentions gender in the constitution (1=yes; 0=no)
228. Nonpregnant and nonnursing women can do the same jobs as men (1=yes; 0=no)
229. Number of maternal deaths
230. Own-account workers, female (% of female employment) (modeled ILO estimate)
231. Own-account workers, male (% of male employment) (modeled ILO estimate)
232. People practicing open defecation (% of population)
233. People practicing open defecation, rural (% of rural population)
234. People practicing open defecation, urban (% of urban population)
235. Persistence to grade 5, female (% of cohort)
236. Persistence to grade 5, male (% of cohort)
237. Population ages 0-14 (% of total)
238. Population ages 0-14, female
239. Population ages 15-64 (% of total)
240. Population ages 15-64, female
241. Population ages 15-64, male
242. Population ages 15-64, total
243. Population ages 65 and above (% of total)
244. Population ages 65 and above, female
245. Population, female
246. Population, female (% of total)
247. Population, total
248. Poverty headcount ratio at $1.90 a day (2011 PPP) (% of population)
249. Poverty headcount ratio at national poverty lines (% of population)
250. Pregnant women receiving prenatal care (%)
251. Prevalence of anemia among women of reproductive age (% of women ages 15-49)
252. Prevalence of HIV, female (% ages 15-24)
253. Prevalence of HIV, male (% ages 15-24)
254. Prevalence of obesity, female (% of female population ages 18+)
255. Prevalence of obesity, male (% of male population ages 18+)
256. Prevalence of overweight, female (% of female adults)
257. Prevalence of overweight, male (% of male adults)
258. Prevalence of overweight, weight for height, female (% of children under 5)
259. Prevalence of overweight, weight for height, male (% of children under 5)
260. Prevalence of severe wasting, weight for height, female (% of children under 5)
261. Prevalence of severe wasting, weight for height, male (% of children under 5)
262. Prevalence of stunting, height for age, female (% of children under 5)
263. Prevalence of stunting, height for age, male (% of children under 5)
264. Prevalence of underweight, weight for age, female (% of children under 5)
265. Prevalence of underweight, weight for age, male (% of children under 5)
266. Prevalence of wasting, weight for height, female (% of children under 5)
267. Prevalence of wasting, weight for height, male (% of children under 5)
268. Primary completion rate, female (% of relevant age group)
269. Primary completion rate, male (% of relevant age group)
270. Primary education, pupils (% female)
271. Primary education, teachers (% female)
272. Probability of Survival to Age 5
273. Probability of Survival to Age 5, Female
274. Probability of Survival to Age 5, Male
275. Progression to secondary school, female (%)
276. Progression to secondary school, male (%)
277. Proportion of seats held by women in national parliaments (%)
278. Proportion of time spent on unpaid domestic and care work, female (% of 24 hour day)
279. Proportion of time spent on unpaid domestic and care work, male (% of 24 hour day)
280. Proportion of women in ministerial level positions (%)
281. Proportion of women subjected to physical and/or sexual violence in the last 12 months (% of women age 15-49)
282. Ratio of female to male labor force participation rate (%) (modeled ILO estimate)
283. Ratio of female to male labor force participation rate (%) (national estimate)
284. Ratio of female to male youth unemployment rate (% ages 15-24) (modeled ILO estimate)
285. Ratio of female to male youth unemployment rate (% ages 15-24) (national estimate)
286. Rural population, female (% of total)
287. Rural population, male (% of total)
288. School enrollment, primary (gross), gender parity index (GPI)
289. School enrollment, primary and secondary (gross), gender parity index (GPI)
290. School enrollment, primary, female (% gross)
291. School enrollment, primary, female (% net)
292. School enrollment, primary, male (% gross)
293. School enrollment, primary, male (% net)
294. School enrollment, secondary (gross), gender parity index (GPI)
295. School enrollment, secondary, female (% gross)
296. School enrollment, secondary, female (% net)
297. School enrollment, secondary, male (% gross)
298. School enrollment, secondary, male (% net)
299. School enrollment, tertiary (gross), gender parity index (GPI)
300. School enrollment, tertiary, female (% gross)
301. School enrollment, tertiary, male (% gross)
302. Secondary education, pupils (% female)
303. Secondary education, teachers (% female)
304. Self-employed, female (% of female employment) (modeled ILO estimate)
305. Self-employed, male (% of male employment) (modeled ILO estimate)
306. Sex ratio at birth (male births per female births)
307. Smoking prevalence, females (% of adults)
308. Smoking prevalence, males (% of adults)
309. Start-up procedures to register a business, female (number)
310. Start-up procedures to register a business, male (number)
311. Suicide mortality rate, female (per 100,000 female population)
312. Suicide mortality rate, male (per 100,000 male population)
313. Survival Rate from Age 15-60
314. Survival Rate from Age 15-60, Female
315. Survival Rate from Age 15-60, Male
316. Tertiary education, academic staff (% female)
317. Time required to start a business, female (days)
318. Time required to start a business, male (days)
319. Time-related underemployment, female (% of employment)
320. Time-related underemployment, male (% of employment)
321. Total alcohol consumption per capita, female (liters of pure alcohol, projected estimates, female 15+ years of age)
322. Total alcohol consumption per capita, male (liters of pure alcohol, projected estimates, male 15+ years of age)
323. Unemployment with advanced education, female (% of female labor force with advanced education)
324. Unemployment with advanced education, male (% of male labor force with advanced education)
325. Unemployment with basic education, female (% of female labor force with basic education)
326. Unemployment with basic education, male (% of male labor force with basic education)
327. Unemployment with intermediate education, female (% of female labor force with intermediate education)
328. Unemployment with intermediate education, male (% of male labor force with intermediate education)
329. Unemployment, female (% of female labor force) (modeled ILO estimate)
330. Unemployment, female (% of female labor force) (national estimate)
331. Unemployment, male (% of male labor force) (modeled ILO estimate)
332. Unemployment, male (% of male labor force) (national estimate)
333. Unemployment, youth female (% of female labor force ages 15-24) (modeled ILO estimate)
334. Unemployment, youth female (% of female labor force ages 15-24) (national estimate)
335. Unemployment, youth male (% of male labor force ages 15-24) (modeled ILO estimate)
336. Unemployment, youth male (% of male labor force ages 15-24) (national estimate)
337. Unmet need for contraception (% of married women ages 15-49)
338. Urban population, female (% of total)
339. Urban population, male (% of total)
340. Vulnerable employment, female (% of female employment) (modeled ILO estimate)
341. Vulnerable employment, male (% of male employment) (modeled ILO estimate)
342. Wage and salaried workers, female (% of female employment) (modeled ILO estimate)
343. Wage and salaried workers, male (% of male employment) (modeled ILO estimate)
344. Wanted fertility rate (births per woman)
345. Woman's testimony carries the same evidentiary weight in court as a man's (1=yes; 0=no)
346. Women making their own informed decisions regarding sexual relations, contraceptive use and reproductive health care (% of women age 15-49)
347. Women participating in decision of visits to family, relatives, friends (% of women age 15-49)
348. Women participating in decision of what food to cook daily (% of women age 15-49)
349. Women participating in making daily purchase decisions (% of women age 15-49)
350. Women participating in making major household purchase decisions (% of women age 15-49)
351. Women participating in none of the three decisions (own health care, major household purchases, and visiting family) (% of women age 15-49)
352. Women participating in own health care decisions (% of women age 15-49)
353. Women participating in the three decisions (own health care, major household purchases, and visiting family) (% of women age 15-49)
354. Women who believe a husband is justified in beating his wife (any of five reasons) (%)
355. Women who believe a husband is justified in beating his wife (any of five reasons) (%): Q1 (lowest)
356. Women who believe a husband is justified in beating his wife (any of five reasons) (%): Q2
357. Women who believe a husband is justified in beating his wife (any of five reasons) (%): Q3
358. Women who believe a husband is justified in beating his wife (any of five reasons) (%): Q4
359. Women who believe a husband is justified in beating his wife (any of five reasons) (%): Q5 (highest)
360. Women who believe a husband is justified in beating his wife when she argues with him (%)
361. Women who believe a husband is justified in beating his wife when she argues with him (%): Q1 (lowest)
362. Women who believe a husband is justified in beating his wife when she argues with him (%): Q2
363. Women who believe a husband is justified in beating his wife when she argues with him (%): Q3
364. Women who believe a husband is justified in beating his wife when she argues with him (%): Q4
365. Women who believe a husband is justified in beating his wife when she argues with him (%): Q5 (highest)
366. Women who believe a husband is justified in beating his wife when she burns the food (%)
367. Women who believe a husband is justified in beating his wife when she burns the food (%): Q1 (lowest)
368. Women who believe a husband is justified in beating his wife when she burns the food (%): Q2
369. Women who believe a husband is justified in beating his wife when she burns the food (%): Q3
370. Women who believe a husband is justified in beating his wife when she burns the food (%): Q4
371. Women who believe a husband is justified in beating his wife when she burns the food (%): Q5 (highest)
372. Women who believe a husband is justified in beating his wife when she goes out without telling him (%)
373. Women who believe a husband is justified in beating his wife when she goes out without telling him (%): Q1 (lowest)
374. Women who believe a husband is justified in beating his wife when she goes out without telling him (%): Q2
375. Women who believe a husband is justified in beating his wife when she goes out without telling him (%): Q3
376. Women who believe a husband is justified in beating his wife when she goes out without telling him (%): Q4
377. Women who believe a husband is justified in beating his wife when she goes out without telling him (%): Q5 (highest)
378. Women who believe a husband is justified in beating his wife when she neglects the children (%)
379. Women who believe a husband is justified in beating his wife when she neglects the children (%): Q1 (lowest)
380. Women who believe a husband is justified in beating his wife when she neglects the children (%): Q2
381. Women who believe a husband is justified in beating his wife when she neglects the children (%): Q3
382. Women who believe a husband is justified in beating his wife when she neglects the children (%): Q4
383. Women who believe a husband is justified in beating his wife when she neglects the children (%): Q5 (highest)
384. Women who believe a husband is justified in beating his wife when she refuses sex with him (%)
385. Women who believe a husband is justified in beating his wife when she refuses sex with him (%): Q1 (lowest)
386. Women who believe a husband is justified in beating his wife when she refuses sex with him (%): Q2
387. Women who believe a husband is justified in beating his wife when she refuses sex with him (%): Q3
388. Women who believe a husband is justified in beating his wife when she refuses sex with him (%): Q4
389. Women who believe a husband is justified in beating his wife when she refuses sex with him (%): Q5 (highest)
390. Women who believe a wife is justified refusing sex with her husband for all of the reasons (%)
391. Women who believe a wife is justified refusing sex with her husband for all of the reasons (%): Q1 (lowest)
392. Women who believe a wife is justified refusing sex with her husband for all of the reasons (%): Q2
393. Women who believe a wife is justified refusing sex with her husband for all of the reasons (%): Q3
394. Women who believe a wife is justified refusing sex with her husband for all of the reasons (%): Q4
395. Women who believe a wife is justified refusing sex with her husband for all of the reasons (%): Q5 (highest)
396. Women who believe a wife is justified refusing sex with her husband for none of the reasons (%)
397. Women who believe a wife is justified refusing sex with her husband for none of the reasons (%): Q1 (lowest)
398. Women who believe a wife is justified refusing sex with her husband for none of the reasons (%): Q2
399. Women who believe a wife is justified refusing sex with her husband for none of the reasons (%): Q3
400. Women who believe a wife is justified refusing sex with her husband for none of the reasons (%): Q4
401. Women who believe a wife is justified refusing sex with her husband for none of the reasons (%): Q5 (highest)
402. Women who believe a wife is justified refusing sex with her husband if she has recently given birth (%)
403. Women who believe a wife is justified refusing sex with her husband if she has recently given birth (%): Q1 (lowest)
404. Women who believe a wife is justified refusing sex with her husband if she has recently given birth (%): Q2
405. Women who believe a wife is justified refusing sex with her husband if she has recently given birth (%): Q3
406. Women who believe a wife is justified refusing sex with her husband if she has recently given birth (%): Q4
407. Women who believe a wife is justified refusing sex with her husband if she has recently given birth (%): Q5 (highest)
408. Age population, age 05, female, interpolated
409. Age population, age 04, male, interpolated
410. Age population, age 04, female, interpolated
411. Age population, age 03, male, interpolated
412. Age population, age 03, female, interpolated
413. Women who believe a wife is justified refusing sex with her husband if she is tired or not in the mood (%)
414. Women who believe a wife is justified refusing sex with her husband if she is tired or not in the mood (%): Q1 (lowest)
415. Age population, age 02, male, interpolated
416. Age population, age 02, female, interpolated
417. Age population, age 01, male, interpolated
418. Age population, age 01, female, interpolated
419. Age population, age 0, male, interpolated
420. Age population, age 0, female, interpolated
421. Age dependency ratio (% of working-age population)
422. Mean age at first marriage, male
423. Mean age at first marriage, female
424. Adolescent fertility rate (births per 1,000 women ages 15-19)
425. Adjusted net enrollment rate, primary, male (% of primary school age children)
426. Adjusted net enrollment rate, primary, female (% of primary school age children)
427. Account ownership at a financial institution or with a mobile-money-service provider, male (% of population ages 15+)
428. Account ownership at a financial institution or with a mobile-money-service provider, female (% of population ages 15+)
429. Antiretroviral therapy coverage (% of adult males living with HIV)
430. Antiretroviral therapy coverage (% of adult females living with HIV)
431. Women who believe a wife is justified refusing sex with her husband if she is tired or not in the mood (%): Q2
432. Women who believe a wife is justified refusing sex with her husband if she is tired or not in the mood (%): Q3
433. Women who believe a wife is justified refusing sex with her husband if she is tired or not in the mood (%): Q4
434. Women who believe a wife is justified refusing sex with her husband if she is tired or not in the mood (%): Q5 (highest)
435. Women who believe a wife is justified refusing sex with her husband if she knows he has sex with other women (%)
436. Women who believe a wife is justified refusing sex with her husband if she knows he has sex with other women (%): Q1 (lowest)
437. Women who believe a wife is justified refusing sex with her husband if she knows he has sex with other women (%): Q2
438. Women who believe a wife is justified refusing sex with her husband if she knows he has sex with other women (%): Q3
439. Women who believe a wife is justified refusing sex with her husband if she knows he has sex with other women (%): Q4
440. Women who believe a wife is justified refusing sex with her husband if she knows he has sex with other women (%): Q5 (highest)
441. Women who believe a wife is justified refusing sex with her husband if she knows he has sexually transmitted disease (%)
442. Women who believe a wife is justified refusing sex with her husband if she knows he has sexually transmitted disease (%): Q1 (lowest)
443. Women who believe a wife is justified refusing sex with her husband if she knows he has sexually transmitted disease (%): Q2
444. Women who believe a wife is justified refusing sex with her husband if she knows he has sexually transmitted disease (%): Q3
445. Women who believe a wife is justified refusing sex with her husband if she knows he has sexually transmitted disease (%): Q4
446. Women who believe a wife is justified refusing sex with her husband if she knows he has sexually transmitted disease (%): Q5 (highest)
447. Women who do not own a house (% of women age 15-49)
448. Women who do not own a house (% of women age 15-49): Q1 (lowest)
449. Women who do not own a house (% of women age 15-49): Q2
450. Women who do not own a house (% of women age 15-49): Q3
451. Women who do not own a house (% of women age 15-49): Q4
452. Women who do not own a house (% of women age 15-49): Q5 (highest)
453. Women who do not own land (% of women age 15-49)
454. Women who do not own land (% of women age 15-49): Q1 (lowest)
455. Women who do not own land (% of women age 15-49): Q2
456. Women who do not own land (% of women age 15-49): Q3
457. Women who do not own land (% of women age 15-49): Q4
458. Women who do not own land (% of women age 15-49): Q5 (highest)
459. Women who own a house alone (% of women age 15-49)
460. Women who own a house alone (% of women age 15-49): Q1 (lowest)
461. Women who own a house alone (% of women age 15-49): Q2
462. Women who own a house alone (% of women age 15-49): Q3
463. Women who own a house alone (% of women age 15-49): Q4
464. Women who own a house alone (% of women age 15-49): Q5 (highest)
465. Women who own a house both alone and jointly (% of women age 15-49)
466. Women who own a house both alone and jointly (% of women age 15-49): Q1 (lowest)
467. Women who own a house both alone and jointly (% of women age 15-49): Q2
468. Women who own a house both alone and jointly (% of women age 15-49): Q3
469. Women who own a house both alone and jointly (% of women age 15-49): Q4
470. Women who own a house both alone and jointly (% of women age 15-49): Q5 (highest)
471. Women who own a house jointly (% of women age 15-49)
472. Women who own a house jointly (% of women age 15-49): Q1 (lowest)
473. Women who own a house jointly (% of women age 15-49): Q2
474. Women who own a house jointly (% of women age 15-49): Q3
475. Women who own a house jointly (% of women age 15-49): Q4
476. Women who own a house jointly (% of women age 15-49): Q5 (highest)
477. Women who own land alone (% of women age 15-49)
478. Women who own land alone (% of women age 15-49): Q1 (lowest)
479. Women who own land alone (% of women age 15-49): Q2
480. Women's share of population ages 15+ living with HIV (%)
481. Women who were first married by age 18 (% of women ages 20-24)
482. Women who were first married by age 15 (% of women ages 20-24)
483. Women who own land jointly (% of women age 15-49): Q5 (highest)
484. Women who own land jointly (% of women age 15-49): Q4
485. Women who own land jointly (% of women age 15-49): Q3
486. Women who own land jointly (% of women age 15-49): Q2
487. Women who own land jointly (% of women age 15-49): Q1 (lowest)
488. Women who own land jointly (% of women age 15-49)
489. Women who own land both alone and jointly (% of women age 15-49): Q5 (highest)
490. Women who own land both alone and jointly (% of women age 15-49): Q4
491. Women who own land both alone and jointly (% of women age 15-49): Q3
492. Women who own land both alone and jointly (% of women age 15-49): Q2
493. Women who own land both alone and jointly (% of women age 15-49): Q1 (lowest)
494. Women who own land both alone and jointly (% of women age 15-49)
495. Women who own land alone (% of women age 15-49): Q5 (highest)
496. Women who own land alone (% of women age 15-49): Q4
497. Women who own land alone (% of women age 15-49): Q3
498. Cost of business start-up procedures, male (% of GNI per capita)
499. Cost of business start-up procedures, female (% of GNI per capita)
500. Contributing family workers, male (% of male employment) (modeled ILO estimate)
501. Contributing family workers, female (% of female employment) (modeled ILO estimate)
502. Contraceptive prevalence, modern methods (% of women ages 15-49)
503. Contraceptive prevalence, any methods (% of women ages 15-49)
504. Completeness of birth registration, male (%)
505. Completeness of birth registration, female (%)
506. Children out of school, primary, male
507. Children out of school, primary, female
508. Children in employment, male (% of male children ages 7-14)
509. Children in employment, female (% of female children ages 7-14)
510. Cause of death, by non-communicable diseases, ages 35-59, male (% relevant age)
511. Cause of death, by non-communicable diseases, ages 35-59, female (% relevant age)
512. Cause of death, by non-communicable diseases, ages 15-34, male (% relevant age)
513. Cause of death, by non-communicable diseases, ages 15-34, female (% relevant age)
514. Cause of death, by injury, ages 35-59, male (% relevant age)
515. Cause of death, by injury, ages 35-59, female (% relevant age)
516. Cause of death, by injury, ages 15-34, male (% relevant age)
517. Cause of death, by injury, ages 15-34, female (% relevant age)
518. Cause of death, by communicable diseases and maternal, prenatal and nutrition conditions, ages 35-59, male (% relevant age)
519. Cause of death, by communicable diseases and maternal, prenatal and nutrition conditions, ages 35-59, female (% relevant age)
520. Cause of death, by communicable diseases and maternal, prenatal and nutrition conditions, ages 15-34, male (% relevant age)
521. Cause of death, by communicable diseases and maternal, prenatal and nutrition conditions, ages 15-34, female (% relevant age)
522. Births attended by skilled health staff (% of total)
523. Birth rate, crude (per 1,000 people)
524. Age population, age 05, male, interpolated

**B: Data dictionary for the indicators selected for final analysis**

| **No.** | **Variable name** | **Indicator name** | **Long definition** | **Data source** |
| --- | --- | --- | --- | --- |
| 1 | Primary education attainment | Educational attainment, at least completed primary, population 25+ years, female (%) (cumulative) | The percentage of population ages 25 and over that attained or completed primary education. | UNESCO Institute for Statistics (http://uis.unesco.org/) |
| 2 | Secondary enrolment – gross | School enrollment, secondary, female (% gross) | Gross enrollment ratio is the ratio of total enrollment, regardless of age, to the population of the age group that officially corresponds to the level of education shown. Secondary education completes the provision of basic education that began at the primary level, and aims at laying the foundations for lifelong learning and human development, by offering more subject- or skill-oriented instruction using more specialized teachers. | UNESCO Institute for Statistics (http://uis.unesco.org/) |
| 3 | Secondary enrolment – net | School enrollment, secondary, female (% net) | Net enrollment rate is the ratio of children of official school age who are enrolled in school to the population of the corresponding official school age. Secondary education completes the provision of basic education that began at the primary level, and aims at laying the foundations for lifelong learning and human development, by offering more subject- or skill-oriented instruction using more specialized teachers. | UNESCO Institute for Statistics (http://uis.unesco.org/) |
| 4 | Progression to secondary school | Progression to secondary school, female (%) | Progression to secondary school refers to the number of new entrants to the first grade of secondary school in a given year as a percentage of the number of students enrolled in the final grade of primary school in the previous year (minus the number of repeaters from the last grade of primary education in the given year). | UNESCO Institute for Statistics (http://uis.unesco.org/) |
| 5 | Post-secondary education attainment | Educational attainment, at least completed post-secondary, population 25+, female (%) (cumulative) | The percentage of population ages 25 and over that attained or completed post-secondary non-tertiary education. | UNESCO Institute for Statistics (http://uis.unesco.org/) |
| 6 | Upper secondary education attainment | Educational attainment, at least completed upper secondary, population 25+, female (%) (cumulative) | The percentage of population ages 25 and over that attained or completed upper secondary education. | UNESCO Institute for Statistics (http://uis.unesco.org/) |
| 7 | Lower secondary education completion | Lower secondary completion rate, female (% of relevant age group) | Lower secondary education completion rate is measured as the gross intake ratio to the last grade of lower secondary education (general and pre-vocational). It is calculated as the number of new entrants in the last grade of lower secondary education, regardless of age, divided by the population at the entrance age for the last grade of lower secondary education. | UNESCO (United Nations Educational, Scientific and Cultural Organization) Institute for Statistics (2018). Data Centre. http://data.uis.unesco.org. Accessed 15 June 2018. |
| 8 | Tertiary school enrolment - gross | Educational attainment, at least completed post-secondary, population 25+, female (%) (cumulative) | The percentage of population ages 25 and over that attained or completed post-secondary non-tertiary education. | UNESCO Institute for Statistics (http://uis.unesco.org/) |
| 9 | Education attainment – short tertiary | Educational attainment, at least completed short-cycle tertiary, population 25+, female (%) (cumulative) | The percentage of population ages 25 and over that attained or completed short-cycle tertiary education. | UNESCO Institute for Statistics (http://uis.unesco.org/) |
| 10 | Education attainment – Bachelors | Educational attainment, at least Bachelor's or equivalent, population 25+, female (%) (cumulative) | The percentage of population ages 25 and over that attained or completed Bachelor's or equivalent. | UNESCO Institute for Statistics (http://uis.unesco.org/) |
| 11 | Education attainment – Masters | Educational attainment, at least Master's or equivalent, population 25+, female (%) (cumulative) | The percentage of population ages 25 and over that attained or completed Master's or equivalent. | UNESCO Institute for Statistics (http://uis.unesco.org/) |
| 12 | Education attainment – Doctoral | Educational attainment, Doctoral or equivalent, population 25+, female (%) (cumulative) | The percentage of population ages 25 and over that attained or completed Doctoral or equivalent. | UNESCO Institute for Statistics (http://uis.unesco.org/) |
| 13 | Firms with female ownership | Firms with female participation in ownership (% of firms) | Firms with female participation in ownership are the percentage of firms with a woman among the principal owners. | World Bank, Enterprise Surveys (http://www.enterprisesurveys.org/). |
| 14 | Houses with female head of house | Female headed households (% of households with a female head) | Female headed households shows the percentage of households with a female head. | Demographic and Health Surveys. |
| 15 | Firms with female in senior or middle management | Female share of employment in senior and middle management (%) | The proportion of females in total employment in senior and middle management. It corresponds to major group 1 in both ISCO-08 and ISCO-88 minus category 14 in ISCO-08 (hospitality, retail and other services managers) and minus category 13 in ISCO-88 (general managers), since these comprise mainly managers of small enterprises. | ILO (International Labour Organization) (2018a). ILOSTAT database. www.ilo.org/ilostat. |
| 16 | Firms with females at top-management | Firms with female top manager (% of firms) | Firms with female top manager refers to the percentage of firms in the private sector who have females as top managers. Top manager refers to the highest ranking manager or CEO of the establishment. This person may be the owner if he/she works as the manager of the firm. The results are based on surveys of more than 100,000 private firms. | World Bank, Enterprise Surveys (http://www.enterprisesurveys.org/). |
| 17 | Child marriage | Child marriage | Child marriage, women married by age 18 (% of women ages 20–24 years who are married or in union) | United Nations Statistics Division (2018a). Global SDG Indicators Database. https://unstats.un.org/sdgs/indicators/database/. Accessed 20 June, 2018. |
| 18 | Married before 18 | Women who were first married by age 18 (% of women ages 20-24) | Women who were first married by age 18 refers to the percentage of women ages 20-24 who were first married by age 18. | Demographic and Health Surveys (DHS), Multiple Indicator Cluster Surveys (MICS), AIDS Indicator Surveys(AIS), Reproductive Health Survey(RHS), and other household surveys. |
| 19 | Married before 15 | Women who were first married by age 15 (% of women ages 20-24) | Women who were first married by age 15 refers to the percentage of women ages 20-24 who were first married by age 15. | Demographic and Health Surveys (DHS) |
| 20 | Wage and Salaried workers | Wage and salaried workers, female (% of female employment) (modeled ILO estimate) | Wage and salaried workers (employees) are those workers who hold the type of jobs defined as "paid employment jobs," where the incumbents hold explicit (written or oral) or implicit employment contracts that give them a basic remuneration that is not directly dependent upon the revenue of the unit for which they work. | International Labour Organization, ILOSTAT database. Data retrieved in April 2019. |
| 21 | Female employers | Employers, female (% of female employment) (modeled ILO estimate) | Employers are those workers who, working on their own account or with one or a few partners, hold the type of jobs defined as a "self-employment jobs" i.e. jobs where the remuneration is directly dependent upon the profits derived from the goods and services produced), and, in this capacity, have engaged, on a continuous basis, one or more persons to work for them as employee(s). | International Labour Organization, ILOSTAT database. Data retrieved in April 2019. |
| 22 | Vulnerable employment | Vulnerable employment, female (% of female employment) (modeled ILO estimate) | Vulnerable employment is contributing family workers and own-account workers as a percentage of total employment. | Derived using data from International Labour Organization, ILOSTAT database. Data retrieved in April 2019. |
| 23 | Female representation in parliament | Proportion of seats held by women in national parliaments (%) | Women in parliaments are the percentage of parliamentary seats in a single or lower chamber held by women. | IPU (Inter-Parliamentary Union). 2018. Women in national parliaments. www.ipu.org/wmn-e/classif-arc.htm. Accessed 24 April 2018. |
| 24 | Intimate partner violence |  | Violence against women ever experienced, intimate partner (% of female population ages 15 and older) | UN Women (United Nations Entity for Gender Equality and the Empowerment of Women). 2018. UN Women Global Database on Violence against Women. New York. http://evaw-global-database.unwomen.org. Accessed 19 April 2018. |
| 25 | Maternal mortality rate |  | Maternal mortality ratio (deaths per 100,000 live births) | UN Maternal Mortality Estimation Group (World Health Organization, United Nations Children’s Fund, United Nations Population Fund and World Bank). 2017. Maternal mortality data. http://data.unicef.org/topic/ maternal-health/maternal-mortality/. Accessed 16 April 2018. |
